# Bioactive Cembranoids from the Soft Coral *Sinularia crassa*

**DOI:** 10.3390/md9101955

**Published:** 2011-10-17

**Authors:** Chih-Hua Chao, Kuei-Ju Chou, Chiung-Yao Huang, Zhi-Hong Wen, Chi-Hsin Hsu, Yang-Chang Wu, Chang-Feng Dai, Jyh-Horng Sheu

**Affiliations:** 1 Department of Marine Biotechnology and Resources, National Sun Yat-sen University, Kaohsiung 804, Taiwan; E-Mails: chaochihhua@hotmail.com (C.-H.C.); jzusmile@hotmail.com (K.-J.C.); betty8575@yahoo.com.tw (C.-Y.H.); wzh@mail.nsysu.edu.tw (Z.-H.W.); hsuch@mail.nsysu.edu.tw (C.-H.H.); 2 Chinese Medicinal Research and Development Center, China Medical University and Hospital, Taichung 404, Taiwan; 3 Asian Pacific Ocean Research Center, National Sun Yat-sen University, Kaohsiung 804, Taiwan; 4 College of Chinese Medicine, China Medical University, Taichung 404, Taiwan; E-Mail: yachwu@mail.cmu.edu.tw; 5 Institute of Oceanography, National Taiwan University, Taipei, Taiwan; E-Mail: corallab@ntu.edu.tw

**Keywords:** *Sinularia crassa*, crassarines A-H, anti-inflammatory

## Abstract

Eight new cembranoids, crassarines A–H (**1**–**8**) were isolated from the Formosan soft coral *Sinularia crassa*. Compounds **1**–**3** represent the rare cembranoids with a 1,12-oxa-bridged tetrahydrofuran ring, while **4** and **5** are the firstly discovered 1,11-oxa-bridged tetrahydropyranocembranoids. The absolute configuration of **6** was determined using the Mosher’s method. Compounds **6** and **8** were found to significantly inhibit the expression of both pro-inflammatory iNOS and COX-2 proteins at 10 μM, respectively, while compounds **4**–**8** were found to be non-cytotoxic toward the selected human liver cancer cells.

## 1. Introduction

**Chart 1:**
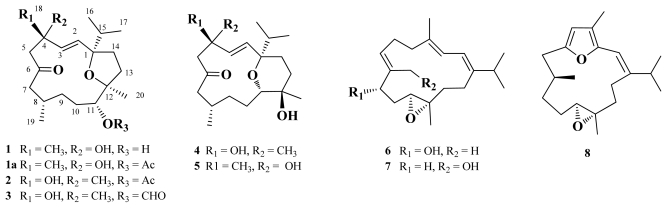
The structures of crassarines A–H (**1**–**8**).

Soft corals were proven to be a rich source of terpenoids [1]. We previously have isolated a series of bioactive cembrane- [2–4] and norcembrane- [5–8] diterpenoids from the Formosan soft corals of the genus *Sinularia*. Although this genus has been well studied regarding bioactive constituents, previous investigations on an Indian soft coral *Sinularia crassa* (Tixier-Durivault, 1951) had resulted in the isolation of only a sphingosine and a steroid possessing anti-inflammatory [9,10] and 5α-reductase inhibitiory activities [11], respectively. This prompted us to investigate the bioactive compounds from the Formosan soft coral *S. crassa* and the present study has led to the isolation of eight new cembranoids, crassarines A–H (**1**–**8**, see Chart 1) from the ethanolic extract of this organism. The structures of these compounds have been established by extensive spectroscopic analysis and chemical method. The anti-inflammatory activity of **1**–**8** to inhibit up-regulation of the pro-inflammatory iNOS (inducible nitric oxide synthase) and COX-2 (cyclooxygenase-2) proteins in LPS (lipopolysaccharide)-stimulated RAW264.7 macrophage cells and the cytotoxicity of compounds **4**–**8** against a panel of cancer cell lines including human liver carcinoma (HepG2 and HepG3), human breast carcinoma (MCF-7 and MDA-MB-231), and human lung carcinoma (A-549) were evaluated in order to discover bioactive natural products.

## 2. Results and Discussion

**Figure 1:**
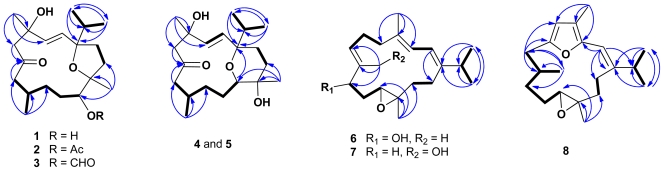
Selected ^1^H–^1^H COSY (**—**) and HMBC (→) correlations of **1**–**8**.

The HRESIMS of crassarine A (**1**) exhibited a pseudomolecular ion peak at *m/z* 361.2353 [M + Na]^+^, consistent with a molecular formula of C_20_H_34_O_4_, appropriate for four degrees of unsaturation. The IR spectrum of **1** showed a broad absorption band at 3461 cm^−1^ and a strong absorption band at 1698 cm^−1^, implying the presence of hydroxy and carbonyl groups. The latter was identified as a ketone functionality from the carbon resonance at *δ* 211.8 (Table 1). In addition, carbon resonances at *δ* 133.3 (CH) and 134.3 (CH) were attributed to the presence of an 1,2-disubstituted double bond. The above functionalities accounted for two of the four degrees of unsaturation, suggesting a bicyclic structure in **1**. By interpretation of ^1^H–^1^H COSY correlations, it was possible to establish three partial structures from both H-7 and H_3_-19 to H-8, H-8 to H-11, H_2_-13 to H_2_-14, and both H_3_-16 and H_3_-17 to H-15. Subsequently, these partial structures were connected by the HMBC correlations (Figure 1). According to the downfield-shifted carbon chemical shifts at *δ* 88.1 (C-1, C), 75.0 (C-11, CH), and 85.7 (C-12, C) [12] as well as the HMBC correlations from H_3_-20 to C-11, C-12, and C-13 and H_3_-16 (or H_3_-17) to C-17 (or C-16), C-15, and C-1, an ether linkage between C-1 and C-12 forming a tetrahydrofuran (THF) ring and a hydroxy group at C-11 were assigned for **1**. The location of C-6 ketone was suggested from the carbon resonances of the adjacent methylenes at *δ* 53.3 (C-5) and 51.6 (C-7). This was further confirmed by the HMBC correlations from both H_2_-7 and H_2_-5 to C-6. In addition, the HMBC correlations from H_3_-18 to C-3, C-4, and C-5 helped to locate the C-2/C-3 double bond and a hydroxy group at quaternary C-4 (*δ* 71.4). Hence, the planar structure of **1**, a cembranoid possessing a 1,12-bridged tetrahydrofuran ring, was established as shown in Figure 1.

**Figure 2:**
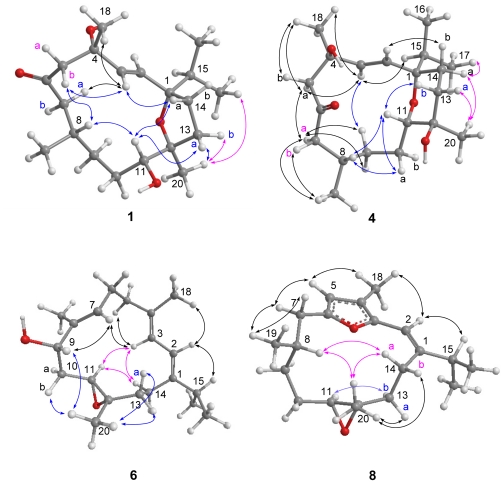
Selected NOE correlations for compounds **1**, **4**, **6**, and **8**.

The *E* geometry for the C-2/C-3 double bond was deduced from a 16.0 Hz coupling constant (Table 1) between H-2 and H-3. The relative configuration of **1** was determined by the interpretation of NOE correlations (Figure 2). The NOE correlations between H_3_-20/H_3_-16 (or H_3_-17), H-11/H-13a (*δ*_H_ 2.61), H-11/H-8, and H_3_-20/H_2_-13 suggested the 1S*,8S*,11R*,12S* configuration as depicted in Figure 2. In addition, the NOE correlations observed for H-2 with both H-15 and H_3_-18 and for H_3_-18 with H-3 suggested the 4S* configuration. In order to understand the orientation of 4-OH and 11-OH, the pyridine-induced solvent shifts were measured [13,14]. The significant differences of chemical shifts (Δ*δ* = *δ* CDCl_3_ – *δ* C_5_D_5_N ) due to pyridine-induced deshielding effect of hydroxy group were observed for H-7a (Δ*δ* = −0.93 ppm), H_3_-20 (Δ*δ* = −0.24 ppm), and H-13a (Δ*δ* = −0.63 ppm) (Table 2), suggesting that 4-OH is close to H-7a, and the 11-OH is not only close to H-13a but also gauche-oriented to H_3_-20, as shown in Figure 2. To determine the absolute configuration, we applied the Mosher’s method on **1**. However, we were unable to prepare the corresponding Mosher esters of **1** by usual reaction conditions [3,4]. This might be due to the steric hindrance of THF ring adjacent to C-11.

HRESIMS analysis of crassarine B (**2**) provided a molecular formula of C_22_H_36_O_5_ ([M + Na]^+^ *m/z* 403.2463). The ^1^H and ^13^C NMR spectroscopic data of **2** were close to those of **1**. A comparison of NMR spectroscopic data of **2** with those of **1** indicated that **2** possesses an acetoxy group [*δ*_C_ 170.9 (C), *δ*_C_ 21.0 (CH_3_); *δ*_H_ 2.09], which was suggested to be attached at C-11 due to the downfield-shifted proton resonance at *δ*_H_ 4.08 (1H, br d, *J* = 10.5 Hz, H-11) in comparison with the relevant case of 11-OH analogue **1** (*δ*_H_ 3.24, 1H, br d, *J* = 9.6 Hz, H-11). The structure elucidation of **2** was accomplished by an extensive analysis of its 2D NMR correlations, which led to the establishment of its planar structure, as shown in Figure 1. Except for the C-11 substituent and the THF ring in both compounds **1** and **2**, the differences were observed for the chemical shifts of protons and carbons around the C-4 asymmetric center, in particular those of H_3_-18 (*δ*_H_ 1.37 and *δ*_C_ 28.9 for **1**; *δ*_H_ 1.25 and *δ*_C_ 29.8 for **2**). These observations suggested that the configuration at C-4 in **2** should be opposite to that in **1**. Moreover, **1** and **2** shared the same NOE correlations around asymmetric centers C-1, C-8, C-11, and C-12. To confirm the above elucidation, **1** was acetylated to obtain **1a**, which displayed different ^1^H NMR spectrum to that of **2** (see Experimental). Consequently, **2** was determined to be the 4-*epi*-11-*O*-acetyl derivative of **1**. The ^13^C and ^1^H NMR spectral data of **3** are very similar to that of **2** (Tables 1 and 2); however, ^1^H NMR spectrum of **3** showed a singlet at *δ* 8.18 which correlates with carbon signal at *δ* 160.9 in the HSQC spectrum, indicating the presence of a formyloxy group at C-11 in **3**. On the basis of the above data, **3** was identified as the 11-O-formyl derivative of **2**. Literature review showed that this is the first cembranoid with a formyloxy group.

Crassarine D (**4**) possesses the same molecular formula as that of **1**. The ^13^C NMR data (Table 1) of **4** were mostly similar to those of **1**, except for those of sp^3^ oxygenated carbons, suggesting that they vary mainly in the heterocyclic ring. The upfield shift for H-11 from *δ* 3.24 (1H, br d, *J* = 9.6 Hz) in **1** to *δ* 3.02 (1H, d, *J* = 8.8 Hz) in **4** indicates that an ether linkage should be located between C-1 and C-11 to form a tetrahydropyran (THP) ring. The HMBC correlation from H-11 to C-1 (*δ* 77.5, C) confirmed the presence of this THP ring in **4**, rather than the THF ring in **1**. The detailed analysis of the correlations observed in the COSY, HMBC, and HSQC spectra further assigned all the spectroscopic data and established the planar structure of **4** (Figure 1). The *E* geometry of C-2/C-3 double bond was also deduced from the coupling constant (16.0 Hz) between H-2 and H-3. NOE correlations between H_3_-20/H-14a, H_3_-17/H-14a, H_3_-20/H-13a, and H-11/H-13b suggested that H-11 is an axial proton and oriented oppositely to H_3_-20. Both H-11 and H-8 were suggested to be positioned on the same face based on the observation of NOE correlations between H-11/H-8, H-8/H-10a, and H-10a/H-11. In addition, H-3 showed NOE correlations with both H_3_-18 and H-15 (Figure 2), revealing that H_3_-18 should be pointed toward the same orientation as that of the isopropyl group. Consequently, the 1S*,4R*,8S*,11S*,12R* configuration was suggested for **4**. Crassarine E (**5**) has the same molecular formula as that of **4**. The ^1^H and ^13^C NMR spectroscopic data as well as the proton coupling patterns of **5** are similar to those of **4**. A comparison of NMR spectroscopic data of **5** with those of **4** showed some differences in chemical shifts for protons and carbons neighboring C-4 and C-8, suggesting that they are epimeric at either C-4 or C-8. The NOE correlation between H_3_-18 and H-2 in **5**, instead of H_3_-18 and H-3 in **4** (Figure 2) suggested that compound **5** is a 4-epimer of **4**.

**Figure 3:**
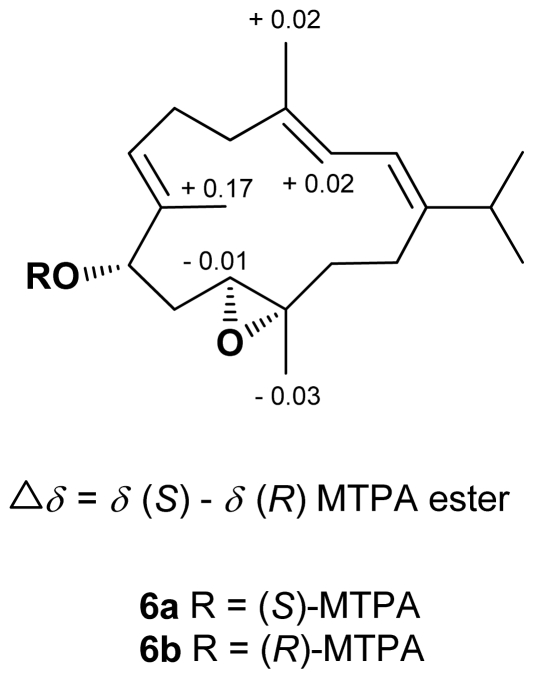
^1^H NMR chemical shift differences of MTPA esters of **6**.

Crassarine F (**6**) was assigned a molecular formula of C_20_H_32_O_2_, according to the HRESIMS and NMR spectroscopic data (Tables 1 and 3). The IR absorption band at 3300 cm^−1^ revealed the presence of hydroxy group. A tetrasubstituted 1,3-butadiene [*δ*_H_ 6.06 (1H, d, *J* = 10.4 Hz) and 5.90 (1H, dd, *J* = 10.4, 1.2 Hz); *δ*_C_ 147.2 (C), 135.4 (C), 121.7 (CH), and 119.1 (CH)], a trisubstituted double bond [*δ*_H_ 5.50 (1H, dd, *J* = 7.2, 6.0 Hz); *δ*_C_ 136.7 (C), and 126.7 (CH)], and a trisubstituted epoxide [*δ*_H_ 2.87 (1H, dd, *J* = 7.6, 6.0 Hz); *δ*_C_ 59.5 (C) and 57.0 (CH)] were also evident. Above NMR signals suggested **6** to be the 1,3-diene cembranoid with an epoxy group [15]. The 11,12-epoxy group was assigned by the HMBC correlations from H_3_-20 to C-11, C-12, and C-13 and H_2_-14 to C-1, C-2, and C-13 (Figure 1). The COSY cross peaks of H_2_-10/H-11 and H_2_-10/H-9 as well as the HMBC correlations from H_3_-19 to C-7, C-8, and C-9 assigned the hydroxy group at C-9 (*δ*_C_ 75.3, CH). These findings and the detailed COSY and HMBC correlations established the planar structure of **6**, as shown in Figure 1. The relative configuration of **6** was determined by the interpretation of NOESY spectrum. The crucial NOE correlations (Figure 2) between H-2/H_3_-18, H-2/H-15, and H-9/H-7 indicated the E geometry for all double bonds and suggested a s-*trans* geometry for the 1,3-diene. NOE correlations between H-11/H-3, H-11/H-14a, and H-3/H-14a showed that these protons should be pointed toward the core of 14-membered ring. Furthermore, the absence of NOE correlation between H-11 and H_3_-20 and the presence of correlation between H-9 and H_3_-20 suggested the 9S*,11S*,12S* configuration, as depicted in Figure 2. The absolute configuration of **6** was determined by the application of Mosher’s method [16,17]. The (*S*)- and (*R*)-MTPA esters of **6** (**6a** and **6b**, respectively) were prepared using the corresponding (*R*)- and (*S*)-MTPA chloride, respectively. The determination of chemical shift differences for the protons neighboring C-9 led to the assignment of the 9*S* configuration in **6** (Figure 3). Thus, the absolute configuration of **6** was determined as 9*S*, 11*S*, 12*S*.

The HRESIMS data of crassarine G (**7**) revealed a molecular formula of C_20_H_32_O_2_, the same as that of **6**. The IR spectrum of **7** disclosed the presence of hydroxy group (ν_max_ 3434 cm^−1^). A comparison of the NMR spectroscopic data of **7** (Tables 1 and 2) with those of **6** revealed that the hydroxy-containing methine (C-9) in **6** was replaced by a sp^3^ methylene in **7**. It was also found that resonances appropriate for H_3_-19 in **6** were absent from the ^1^H and ^13^C NMR spectra of **7** and replaced by signals for a hydroxymethyl group [*δ*_H_ 3.93 and 3.89 (each 1H, d, *J* = 12.0 Hz); *δ*_C_ 59.4 (CH_2_)]. Careful inspection of the 2D NMR spectra of **7** confirmed the above elucidation.

The HRESIMS and ^13^C NMR spectroscopic data of crassarine H (**8**) established a molecular formula of C_20_H_30_O_2_ and six degrees of unsaturation. The ^13^C NMR spectrum showed the presence of a trisubstituted double bond [*δ*_C_ 146.2 (C) and 107.7 (CH)] and a trisubstituted epoxide [*δ*_C_ 65.4 (CH) and 60.7 (C)]. In addition, the carbon resonances at *δ*_C_ 9.1 (CH_3,_ C-18), 151.1 (C, C-6), 146.8 (C, C-3), 109.6 (CH, C-5), and 117.0 (C, C-4) are attributed to the presence of a 2,5-dialkyl-3-methylfuran [18]. This furan moiety and the trisubstituted double bond were found to be conjugated according to the downfield-shifted proton resonance of H-2 at *δ* 5.95 (1H, s) [18]. This was further confirmed by the HMBC correlations from H-2 to C-1, C-3, C-14, and C-15, H_3_-18 to C-3, C-4, and C-5, and H-5 to C-3, C-4, and C-6. The above data together with the detailed inspection of the COSY and HMBC correlations of **8** established its planar structure (Figure 1). The relative configuration of **8** was determined mainly by the assistance of the NOESY experiment. The key NOE correlations between H-2 and both H-15 and H_3_-18 indicated an *E* geometry of C-1/C-2 double bond (Figure 2). The *trans* epoxy group was deduced by the NOE correlations between H-11/H-13b and H_3_-20/H-13a. In addition, H-8 showed an NOE correlation with H_3_-20, instead of H-11, suggesting the 8S*,11S*,12S* configuration for **8**.

**Figure 4:**
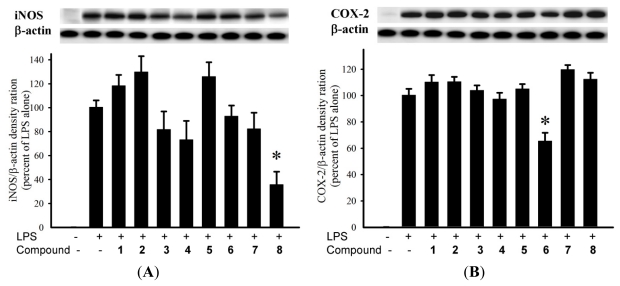
Effect of compounds **1**–**8** at 110 μM on the LPS-induced pro-inflammatory iNOS and on COX-2 protein expression of RAAW264.7 macrophage cells by immunoblot analysis. (**A**) Immunoblots for iNOS and β-actin, and relative density of iNOS; (**B**) Immunoblots for COX-2 and *β*-actin, and relative density of COX-2. The values are means ± SEM (*n* = 6). The relative intensity of the LPS alone stimulated group was taken as 100%. Under the same experimental conditions, 10 μM CAPE (caffeic acid phenethyl ester; Sigma Chemical Company, St. Louis, MO, USA) reduced the levels of the iNOS and COX-2 protein to 0.8 ± 4.5% and 75.6 ± 12.2%, respectively, relative to the control cells stimulated with LPS. * Significantly different from lipopolysaccharide (LPS) alone stimulated group (*P* < 0.05).

The anti-inflammatory activity of diterpenoids **1**–**8** against the accumulation of pro-inflammatory iNOS and COX-2 proteins in RAW264.7 macrophage cells stimulated with LPS was evaluated using immunoblot analysis. At a concentration of 10 μM (Figure 4), **8** was found to significantly reduce the levels of iNOS protein (35.8 ± 10.7%), compared with the control cells stimulated with LPS only. At the same concentration, **6** could reduce COX-2 expression (65.6 ± 6.2%) by LPS treatment. Cytotoxicity of diterpenoids **4**–**8** against HepG2, HepG3, MCF-7, MDA-MB-231, and A-549 cancer cell lines was also evaluated. The results showed that the tested compounds were found to be inactive (IC_50_ > 20 μM) toward the above cancer cell lines after 72 h exposure.

## 3. Experimental Section

### 3.1. General Experimental Procedures

The melting point was determined using a Fisher-Johns melting point apparatus. Optical rotations were determined with a JASCO P1020 digital polarimeter. IR spectrum was obtained on a JASCO FT/IR-4100 spectrophotometer. The NMR spectra were recorded on a Bruker AVANCE 300 FT-NMR (or Varian 400 MR NMR/Varian Unity INOVA 500 FT-NMR) instrument at 300 MHz (or 400/500 MHz) for ^1^H (referenced to TMS, *δ*_H_ 0.00 ppm, for both CDCl_3_ and C_5_D_5_N and 7.15 ppm for C_6_D_6_) and 75 MHz (or 100/125 MHz) for ^13^C (referenced to *δ*_C_ 77.0 for CDCl_3_, to 128.0 ppm for C_6_D_6_, and to internal TMS at *δ*_C_ 0.0 ppm for C_5_D_5_N). ESIMS were recorded by ESI FT-MS on a Bruker APEX II mass spectrometer. Silica gel 60 (Merck, 230–400 mesh) and LiChroprep RP-18 (Merck, 40–63 μm) were used for column chromatography. Precoated silica gel plates (Merck, Kieselgel 60 F254, 0.25 mm) and precoated RP-18 F254S plates (Merck, 1.05560) were used for TLC analyses. High-performance liquid chromatography (HPLC) was performed on a Hitachi L-7100 pump equipped with a Hitachi L-7400 UV detector at 210 nm and a semi-preparative reversed-phase column (Merck, Hibar Purospher RP-18e, 5 μm, 250 × 10 mm).

### 3.2. Animal Material

The soft coral *Sinularia crassa* was collected by hand using scuba off the coast of Sansiantai, Taitung county, Taiwan, in July 2008, at a depth of 10 m, and was stored in a freezer (−20 °C). This soft coral was identified by one of the authors (C.-F.D.). A voucher specimen (Specimen No. SST-03) was deposited in the Department of Marine Biotechnology and Resources, National Sun Yat-sen University.

### 3.3. Extraction and Isolation

The frozen bodies of *S. crassa* (1.1 kg fresh wt) were minced and extracted with EtOH (3 × 2 L, each for 1 day) at room temperature. The organic extract was concentrated to an aqueous suspension and was further partitioned between EtOAc and H_2_O. The EtOAc extract (17.0 g) was fractionated by open column chromatography on silica gel using *n*-hexane-EtOAc and EtOAc-MeOH mixtures of increasing polarity to yield 32 fractions. Fraction 19, eluting with *n*-hexane–EtOAc (5:1), was further separated by silica gel column chromatography with gradient elution (*n*-hexane-EtOAc, 24:1 to 0:1) and followed by RP-18 open column (MeOH-H_2_O, 50% to 100%) to yield three subfractions (19A–19C). Subfraction 19A was subjected to RP-18 HPLC (MeOH-H_2_O, 90%) to obtain compound **8** (2.2 mg). Similarly, compounds **2** (1.1 mg) and **3** (1.0 mg) were obtained from subfraction 19C using RP-18 HPLC (MeOH-H_2_O, 75%). Subfraction 19B was fractionated over silica gel using gradient elution (*n*-hexane-EtOAc, 24:1 to 0:1) to yield three subfractions (19B-1–19B-3). Compounds **4** (3.4 mg) and **5** (2.3 mg) were obtained from subfractions 19B-1 and 19B-2, respectively, using RP-18 HPLC (MeOH-H_2_O, 66%). Subfraction 19B-3 was subjected to normal phase HPLC (*n*-hexane-EtOAc, 2:1) to obtain **1** (2.3 mg). Fractions 22 to 24, eluting with *n*-hexane–EtOAc (1:1), were combined and further separated over silica gel column chromatography (*n*-hexane–EtOAc, gradient elution, 18:1 to 0:1) to give a residue containing terpenoids. This residue was separated over RP-18 column chromatography using gradient elution (MeOH-H_2_O, 50% to 100%) to obtain two subfractions (23A and 23B). Subfraction 23A was further purified by RP-18 HPLC (MeOH-H_2_O, 75%) to yield compound **6** (1.8 mg). In the same manner, compound **7** (8.7 mg) was obtained from subfraction 23B using RP-18 HPLC (MeOH-H_2_O, 80%).

Crassarine A (**1**): colorless oil; [α]^24^ _D_ –93(*c* 0.20, CHCl_3_); IR (KBr) ν_max_ 3461, 2963, 2928, 2873, 1698, 1455, 1380 cm^−1; 1^H NMR and ^13^C NMR data, see Tables 1 and 2; ESIMS *m*/*z* 361 [M + Na]^+^; HRESIMS *m*/*z* 361.2353 [M + Na]^+^ (calcd for C_20_H_34_O_4_Na, 361.2355).

Crassarine B (**2**): colorless oil; [α]^24^ _D_ –13 (*c* 0.11, CHCl_3_); IR (KBr) ν_max_ 3288, 2957, 2925, 2855, 1732, 1698, 1453, 1372, 1237 cm^−1; 1^H NMR and ^13^C NMR data, Tables 1 and 2; ESIMS *m*/*z* 403 [M + Na]^+^; HRESIMS *m*/*z* 403.2463 [M + Na]^+^ (calcd for C_22_H_36_O_5_Na, 403.2460).

Crassarine C (**3**): colorless oil; [α]^24^ _D_ –45 (*c* 0.10, CHCl_3_); IR (KBr) ν_max_ 3483, 2955, 2925, 2855, 1725, 1698, 1455, 1375, 1171 cm^−1; 1^H NMR and ^13^C NMR data, Tables 1 and 2; ESIMS *m*/*z* 389 [M + Na]^+^; HRESIMS *m*/*z* 389.2302 [M + Na]^+^ (calcd for C_21_H_34_O_5_Na, 389.2304).

Crassarine D (**4**): colorless oil; [α]^24^ _D_ –48 (*c* 0.34, CHCl_3_); IR (KBr) ν_max_ 3386, 2955, 2925, 2855, 1716, 1458, 1268, 1036 cm^−1; 1^H NMR and ^13^C NMR data, Tables 1 and 3; ESIMS *m*/*z* 361 [M + Na]^+^; HRESIMS *m*/*z* 361.2354 [M + Na]^+^ (calcd for C_20_H_34_O_4_Na, 361.2355).

Crassarine E (**5**): colorless oil; [α]^24^ _D_ –27 (*c* 0.23, CHCl_3_); IR (KBr) ν_max_ 3453, 2957, 2925, 2855, 1713, 1458, 1261, 1044 cm^−1; 1^H NMR and ^13^C NMR data, Tables 1 and 3; ESIMS *m*/*z* 361 [M + Na]^+^; HRESIMS *m*/*z* 361.2357 [M + Na]^+^ (calcd for C_20_H_34_O_4_Na, 361.2355).

Crassarine F (**6**): colorless oil; [α]^24^ _D_ –63 (*c* 0.18, CHCl_3_); IR (KBr) ν_max_ 3300, 2960, 2926, 2857, 1668, 1458, 1380, 1255, 1036 cm^−1; 1^H NMR and ^13^C NMR data, Tables 1 and 3; ESIMS *m*/*z* 327 [M + Na]^+^; HRESIMS *m*/*z* 327.2302 [M + Na]^+^ (calcd for C_20_H_32_O_2_Na, 327.2300).

Crassarine G (**7**): colorless oil; [α]^24^ _D_ –41 (*c* 0.73, CHCl_3_); IR (KBr) ν_max_ 3434, 2959, 2928, 2872, 1671, 1459, 1383, 1011 cm^−1; 1^H NMR and ^13^C NMR data, Tables 1 and 3; ESIMS *m*/*z* 327 [M + Na]^+^; HRESIMS *m*/*z* 327.2302 [M + Na]^+^ (calcd for C_20_H_32_O_2_Na, 327.2300).

Crassarine H (**8**): colorless oil; [α]^24^ _D_ –12 (*c* 0.22, CHCl_3_); IR (KBr) ν_max_ 2955, 2922, 2855, 1458, 1380 cm^−1; 1^H NMR and ^13^C NMR data, Tables 1 and 2; ESIMS *m*/*z* 325 [M + Na]^+^; HRESIMS *m*/*z* 325.2145 [M + Na]^+^ (calcd for C_20_H_30_O_2_Na, 325.2143).

### *3.4. Acetylation of* **1**

To a stirring solution of compound **1** (0.1 mg) in pyridine (1 mL) was successively added excess acetic acid anhydrous (0.2 mL). After the mixture was stirred over night at room temperature, H_2_O (0.3 mL) was added, and this mixture was subsequently extracted with EtOAc (5 × 6 mL). The combined EtOAc extract was successively washed with saturated aqueous NaHCO_3_ and brine. The organic layer was dried over anhydrous Na_2_SO_4_ and concentrated to give a residue, which was chromatographed on silica gel with *n*-hexane-EtOAc (2:1) as eluent to afford **1a** (0.1 mg) which showed a [M + Na]^+^ peak at *m*/*z* 403 in ESIMS spectrum. Selected ^1^H NMR (CDCl_3_, 300 MHz) spectrum of **1a**: δ 5.89 (1H, d, *J* = 15.9 Hz, H-2 or H-3), 5.77 (1H, d, *J* = 15.9 Hz, H-2 or H-3), 4.83 (1H, br d, *J* = 9.9 Hz, H-11), 2.95 (1H, d, *J* = 15.0 Hz, H-5a), 2.46–2.56 (2H, m, H-5b, H-7a), 2.08 (3H, s, OCOCH_3_), 1.37 (3H, s, H_3_-18), 1.20 (3H, s, H_3_-18), 0.85–0.89 (9H, overlapped, H_3_-19, H_3_-16, and H_3_-17).

### *3.5. Preparation of (*S*)- and (*R*)-MTPA Esters of* **6**

To a solution of **6** (0.5 mg) in pyridine (0.4 mL) was added (*R*)**-**MTPA chloride (25 μL), and the mixture was allowed to stand for 3 h at room temperature. The reaction was quenched by the addition of 1.0 mL of H_2_O, and the mixture was subsequently extracted with EtOAc (3 × 1.0 mL). The EtOAc layers were combined, dried over anhydrous MgSO_4_, and evaporated. The residue was subjected to short silica gel column chromatography using *n*-hexane-EtOAc (8:1) to yield the (*S*)-MTPA ester, **6a** (0.3 mg). The same procedure was used to prepare the (*R*)-MTPA ester, **6b** (0.4 mg from 0.5 mg of **1**), with (*S*)-MTPA chloride. Selected ^1^H NMR (CDCl_3_, 300 MHz) of **6a**: δ 7.38–7.50 (5H, m, Ph), 6.14 (1H, d, *J* = 11.4 Hz, H-2), 6.00 (1H, d, *J* = 11.4 Hz, H-3), 5.61–5.71 (2H, overlapped, H-7 and H-9 ), 3.69 (1H, d, *J* = 12.0 Hz, H-11), 3.56 (3H, s, OMe), 1.80 (3H, s, H_3_-18), 1.39 (3H, s, H_3_-19), 1.10 (3H, s, H_3_-20), 1.07 (3H, d, *J* = 6.9 Hz, H_3_-16 or H_3_-17), 1.03 (3H, d, *J* = 6.9 Hz, H_3_-16 or H_3_-17); selected ^1^H NMR (CDCl_3_, 300 MHz) of **6b**: δ 7.38–7.50 (5H, m, Ph), 6.13 (1H, d, *J* = 11.4 Hz, H-2), 5.98 (1H, d, *J* = 11.4 Hz, H-3), 5.67–5.78 (2H, overlapped, H-7 and H-9), 3.70 (1H, d, *J* = 10.2 Hz, H-11), 3.52 (3H, s, OMe) 1.78 (3H, s, H_3_-18), 1.22 (3H, s, H_3_-19), 1.13 (3H, s, H_3_-20), 1.12 (3H, d, *J* = 6.9 Hz, H_3_-16 or H_3_-17), 1.03 (3H, d, *J* = 6.7 Hz, H_3_-16 or H_3_-17).

### 3.6. Cytotoxicity Testing

Compounds were assayed for cytotoxicity against human liver carcinoma (HepG2 and HepG3), human breast carcinoma (MCF-7 and MDA-MB-231), and human lung carcinoma (A-549) cells using the MTT [3-(4,5-dimethylthiazol-2-yl)-2,5-diphenyltetrazolium bromide] method [19]. Freshly trypsinized cell suspensions were seeded in 96-well microtiter plates at densities of 5000–10,000 cells per well with tested compounds added from DMSO-diluted stock. After 3 days in culture, attached cells were incubated with MTT (0.5 mg/mL, 1 h) and subsequently dissolved in DMSO. The absorbency at 550 nm was then measured using a microplate reader. The IC_50_ is the concentration of agent that reduced cell growth by 50% under the experimental conditions.

### 3.7. *In Vitro* Anti-Inflammatory Assay

Macrophage (RAW264.7) cell line was purchased from ATCC. *In vitro* anti-inflammatory activities of tested compounds were measured by examining the inhibition of LPS induced upregulation of iNOS and COX-2 proteins in macrophage cells using western blotting analysis [20,21].

## 4. Conclusions

Cembranoids with a 1,12-oxa-bridged THF ring, such as compounds **1**–**3**, are rare in natural products. Incensole [22], incensole oxide [23], and incensole acetate [24] are the cembranoids of this class which were isolated from frankincense, the resin produced by the plant Boswellia carteri. It is also noteworthy that the formyloxyl cembranoid, such as **3**, and the 1,11-oxa-bridged tetrahydropyranocembranoids, such as **4** and **5**, were discovered for the first time.

## Figures and Tables

**Table 1:** ^13^C NMR spectroscopic data of compounds **1**–**8**.

#	1 a	1 b	2 c	3 a	4 a	5 a	6 d	7 d	8 d
1	88.1	87.6	88.6	88.8	77.5	77.7	147.2	147.7	146.2
2	133.3	133.8	133.4	133.2	131.6	130.8	119.1	118.6	107.7
3	134.3	135.1	136.4	136.5	139.0	138.3	121.7	122.9	146.8
4	71.4	70.7	72.4	72.4	73.4	71.7	135.4	134.8	117.0
5	53.3	56.4	52.7	52.7	54.0	50.8	38.5	39.4	109.6
6	211.8	209.5	212.9	213.0	215.2	215.7	25.2	25.5	151.1
7	51.6	49.4	51.1	51.2	53.1	54.2	126.7	130.1	35.3
8	28.9	25.8	26.4	26.4	30.8	28.5	136.7	138.0	30.4
9	32.5	32.7	32.9	33.0	32.4	29.7	75.3	33.7	30.2
10	29.4	26.5	26.8	26.9	26.0	24.4	32.3	25.5	24.8
11	75.0	71.1	77.0	77.0	76.2	74.7	57.0	59.1	65.4
12	85.7	86.4	84.7	84.7	70.0	70.1	59.5	60.3	60.7
13	35.2	36.7	34.6	34.4	37.1	36.9	36.4	35.4	40.5
14	30.9	30.4	31.7	31.9	28.4	28.8	24.3	24.1	24.2
15	37.7	38.0	38.6	38.5	40.2	40.3	34.4	33.5	35.2
16	18.0	18.3	18.2	18.2	17.3	17.2	22.5	22.3	21.6
17	17.7	17.8	17.6	17.5	16.8	16.8	22.3	22.7	21.1
18	28.9	31.1	29.8	29.7	28.9	24.5	17.3	16.8	9.1
19	22.6	22.1	22.3	22.3	22.0	20.7	11.7	59.4	20.0
20	23.4	20.8	23.5	24.0	18.8	19.5	18.5	19.0	15.2
OAc			170.9						
			21.0						
CHO				160.9					

a Spectra were measured in CDCl_3_ (100 MHz);

b Spectra were measured in pyridine-*d*_5_ (100 MHz);

c Spectra were measured in CDCl_3_ (125 MHz);

d Spectra were measured in C_6_D_6_ (100 MHz).

**Table 2:** ^1^H NMR Spectroscopic Data of Compounds **1**–**3** and **8**.

#	1, *δ*_H_ (*J* in Hz) a	1, *δ*_H_ (*J* in Hz) b	2, *δ*_H_ (*J* in Hz) c	3, *δ*_H_ (*J* in Hz) a	8, *δ*_H_ (*J* in Hz) d
2	5.73, s	6.28, d (16.0)	5.75, s	5.74, s	5.95, s
3	5.73, s	6.04, d (16.0)	5.75, s	5.74, s	
5	a: 2.79, d (15.6)	a: 2.98, d (13.0)	a: 2.89, d (15.0)	a: 2.89, d (15.0)	5.73,s
	b: 2.61, d (15.6)	b: 2.87, d (13.0)	b: 2.48, d (15.0)	b: 2.48, d (15.0)	
7	a: 2.45, dd (15.6, 8.4)	a: 3.38, dd (16.0, 4.0)	a: 2.52, dd (18.0, 8.5)	a: 2.49, dd (18.0, 8.5)	a: 2.44, br d (12.4)
	b: 2.23, dd (15.6, 5.2)	b: 2.04, dd (16.0, 9.6)	b: 2.16, dd (18.0, 4.0)	b: 2.18, dd (18.0, 4.0)	b: 2.02, m
8	2.02, m	2.41, m	2.29, m	2.29, m	1.96, m
9	1.46, m	1.30, m	1.37, m	1.38, m	1.30, m
			0.97, m	0.99, m	0.93, m
10	a: 1.56, m	a: 2.18, m	a: 1.44, m	a: 1.48, m	a: 1.82, m
	b: 1.25, m	b: 1.63, m	b: 1.38, m	b: 1.37, m	b: 1.20, m
11	3.24, br d (9.6)	3.76, d (10.4)	4.80, br d (10.5)	4.90, br d (8.4)	2.36, dd (10.0, 2.0)
13	a: 1.98, m	a: 2.61, ddd (12.4, 8.4, 2.4)	a: 1.80, m	a: 1.84, m	a: 2.40, m
	b: 1.68, m	b: 1.75, m	b: 1.60, m	b: 1.64, m	b: 1.04, m
14	a: 1.96, m	a: 2.12, m	a: 1.98, m	a: 2.01, m	a: 3.55, dd (12.4, 9.2)
	b: 1.89, m	b: 1.88, m	b: 1.87, m	b: 1.86, m	b: 2.02, m
15	1.76, m	1.81, m	1.75, m	1.75, m	2.22, m
16	0.87, d (6.8)	0.92, d (6.8)	0.86, d (6.8)	0.86, d (6.8)	1.00, d (6.0)
17	0.86, d (6.8)	0.92, d (6.8)	0.84, d (6.8)	0.84, d (6.8)	1.04, d (6.0)
18	1.37, s	1.61, s	1.25, s	1.25, s	1.88, s
19	0.98, d (6.4)	0.94, d (6.8)	0.91, d (6.4)	0.92, d (6.8)	0.82, d (6.4)
20	1.25, s	1.49, s	1.15, s	1.18, s	1.23, s
OAc			2.09, s		
CHO				8.18,s	
4-OH			4.45, s	4.47, s	

a Spectra were measured in CDCl_3_ (400 MHz);

b Spectra were measured in pyridine-*d*_5_ (400 MHz);

c Spectra were measured in CDCl_3_ (500 MHz);

d Spectra were measured in C_6_D_6_ (400 MHz).

**Table 3:** ^1^H NMR Spectroscopic Data of Compounds **4**–**7**.

#	4 a, *δ*_H_ (*J* in Hz)	5 a, *δ*_H_ (*J* in Hz)	6 b, *δ*_H_ (*J* in Hz)	7 b, *δ*_H_ (*J* in Hz)
2	5.81, d (16.0)	5.58, d (16.0)	6.06, d (10.4)	6.08, d (10.8)
3	5.89, d (16.0)	6.07, d (16.0)	5.90, dd (10.4, 1.2)	6.02, d (10.8)
5	a: 2.80, d (16.0)	a: 3.01, d (16.6)	2.04, m	2.00, m
	b: 2.72, d (16.0)	b: 2.41, d (16.6)		
7	a: 2.39, dd (13.6, 11.2)	a: 2.46, dd (11.6, 2.8)	2.10, m	a: 2.13, m
	b: 2.16, dd (13.6, 2.4)	b: 2.07, dd (12.0, 11.6)		b: 2.00, m
8	1.92, m	1.96, m	5.50, dd (7.2, 6.0)	5.26, dd (9.2, 5.2)
9	a: 1.32, m	a: 1.56, m	4.00, dd (8.0, 3.2)	a: 2.36, m
	b: 1.18, m	b: 0.99, m		b: 2.29, m
10	a: 1.49, m	a: 1.57, m	a: 1.99, m	a: 1.72, m
	b: 1.19, m	b: 1.26, m	b: 1.67, m	b: 1.64, m
11	3.02, d (8.8)	3.19, d (10.4)	2.87, dd (7.6, 6.0)	3.00, dd (6.8, 5.2)
13	a: 1.74, m	a: 1.72, m	a: 1.85, m	a: 1.91, m
	b: 1.57, m	b: 1.51, m	b: 1.52, m	b: 1.62, m
14	a: 1.68, m	a: 1.65, m	a: 2.23, m	a: 2.40, m
	b: 1.59, m	b: 1.59, m	b: 1.92, m	b: 1.90, m
15	1.77, m	1.80, m	2.16, m	2.21, m
16	0.78, d (6.8)	0.80, d (7.0)	0.99, d (6.8)	1.00, d (6.8)
17	0.91, d (6.8)	0.90, d (7.0)	0.99, d (6.8)	0.99, d (6.8)
#	**4**^a^, *δ*_H_ (*J* in Hz)	**5**^a^, *δ*_H_ (*J* in Hz)	**6**^b^, *δ*_H_ (*J* in Hz)	**7**^b^, *δ*_H_ (*J* in Hz)
18	1.37, s	1.38, s	1.65, s	1.63, s
19	0.98, d (6.4)	1.00, d (6.4)	1.40, s	3.93, d (12.0)
				3.89, d (12.0)
20	1.11, s	1.15, s	1.12, s	1.15, s

a Spectra were measured in CDCl_3_ (400 MHz);

b Spectra were measured in C_6_D_6_ (400 MHz).
